# Preparing the next generation of genomicists: a laboratory-style course in medical genomics

**DOI:** 10.1186/s12920-015-0124-y

**Published:** 2015-08-12

**Authors:** Michael D. Linderman, Ali Bashir, George A. Diaz, Andrew Kasarskis, Saskia C. Sanderson, Randi E. Zinberg, Milind Mahajan, Hardik Shah, Sabrina Suckiel, Micol Zweig, Eric E. Schadt

**Affiliations:** Icahn Institute for Genomics and Multiscale Biology, Icahn School of Medicine at Mount Sinai, One Gustave L. Levy Place, Box 1498, New York, NY 10029 USA; Department of Genetics and Genomic Sciences, Icahn School of Medicine at Mount Sinai, One Gustave L. Levy Place, New York, NY 10029 USA

**Keywords:** Genomics, Education, Whole genome sequencing

## Abstract

The growing gap between the demand for genome sequencing and the supply of trained genomics professionals is creating an acute need to develop more effective genomics education. In response we developed “Practical Analysis of Your Personal Genome”, a novel laboratory-style medical genomics course in which students have the opportunity to obtain and analyze their own whole genome. This report describes our motivations for and the content of a “practical” genomics course that incorporates personal genome sequencing and the lessons we learned during the first three iterations of this course.

**Electronic supplementary material:**

The online version of this article (doi:10.1186/s12920-015-0124-y) contains supplementary material, which is available to authorized users.

## Introduction

There is an acute need for more effective genomics education for healthcare providers and research scientists [1–6]. Academic institutions are responding with new courses and training programs focused on cutting-edge genomics technologies and their applications to personalized medicine [7–9]. Each of these offerings is different, reflecting the lively debate among and within academic centers about what material to teach, to whom and how to do so most effectively [10].

Despite the current uncertainty about the eventual practice model for genomics and personalized medicine, clinicians, nurses, pharmacists and other healthcare providers must begin preparing for a future where they encounter genomics data in routine practice [11]. We believe this preparation begins by training the genetic counselors, laboratory and medical geneticists and research scientists who will be responsible for translating genomic research into genomic medicine.

The genetics clinic has already become a genomics clinic. Large next-generation sequencing (NGS)-backed test panels and whole exome/genome sequencing (WES/WGS) are producing more, and more ambiguous, findings to be further interpreted by the ordering clinician. These technologies are blurring the traditional roles of the laboratory geneticists who perform the test and the providers who order the test and act on the results. Concurrently, evolving expectations around the return of personal genetic results to study participants [12, 13] will require researchers to develop greater expertise in personal genome analysis.

In response to these trends, starting in 2012, the authors, a multi-disciplinary group of faculty at the Icahn School of Medicine at Mount Sinai (ISMMS), have collaborated to develop and offer a laboratory-style medical genomics course entitled “Practical Analysis of Your Personal Genome” (PAPG) that incorporates personal genome sequencing (PGS). PAPG’s purpose is to prepare the next generation of genetics professionals to directly confront and master the larger scope, scale and complexity of NGS, and specifically WGS. PAPG enrolls 20–25 students per year; it is part of the genetic counseling core curriculum and offered as an elective to medical genetics residents, pathology fellows, medical and graduate students. The enrollment by student background is listed in the Additional file 1.

A detailed description of the objectives and organization for PAPG, course syllabi and lessons learned, including the unexpectedly high uptake of sequencing by students, the challenges faced by students with limited informatics backgrounds in working at “genome scale” and the challenges of maintaining the distinction between genomics education and genome interpretation, are included in the Additional file 1. The remainder of this report describes our motivation for incorporating PGS into PAPG, summarizes the outcomes observed so far, and briefly outlines next steps.

## Student, sequence thyself?

PAPG students were offered the opportunity to obtain and analyze their own whole genome as part of the course at no cost to them (funded by ISMMS) or to use an anonymous reference genome. We hypothesized that for those students who desire to do so the opportunity to apply their knowledge to their own genome would increase motivation, engagement and ultimately improve educational outcomes. That hypothesis was motivated by previous reports of integrating personal genotyping (PGT) into clinical education [9, 14] and further motivated by subsequent reports from Stanford [7], which showed that students who chose PGT had better scores on a genomic knowledge exam than students who did not.

In contrast to courses that incorporate genotyping [7–9], typically from direct-to-consumer providers, PAPG students receive the raw sequencing data and perform the analysis themselves. Thus PAPG students are not receiving genetic findings; instead they are educated on the techniques to generate such findings. Any potential benefits, then, that may result from working with their own genome are applied to the entire analysis process not just to the experience of receiving genetic findings.

There are potential risks associated with accessing personal genetic information, particularly in an educational context [8, 15–17] where the use of genetic information must enhance and not adversely affect learning. The course and sequencing protocol (described in detail in the Additional file 1), which included a pre-requisite course, providing access to no-cost genetic counseling and blinding the instructors to a student’s choice, were designed to mitigate the risk of coercion to use their own genome from peers or instructors, maintain the privacy of the student’s choice and genomic data, facilitate informed decision-making and mitigate the risk of test-related distress while maximizing the additional pedagogical value of incorporating personal WGS.

A companion research study has evaluated the education and psychosocial impact of incorporating personal genomes into genomics education. This manuscript complements previously reported results from the 2012 cohort [18, 19]; interested readers are pointed to those reports for a description of the study methods and findings. The Mount Sinai IRB approved the companion study. The approval process for the study and the course itself are described in more detail in the Additional file 1.

The qualitative evidence from the 2012 cohort suggests that the opportunity to analyze one’s own genome, as hypothesized, positively contributed to student motivation and engagement with most, although not all, students reporting low levels of test-related distress and decisional regret [19]. Students further reported that they were more persistent in overcoming the practical challenges of genome analysis and had a better understanding of the patient experience as a result of working with their own genome. Similar results were observed in the 2013 and 2014 cohorts.

However, we can’t quantitatively determine whether analyzing your own genome improves educational outcomes. The context of the courses did not permit the companion research study to be structured as a randomized trial with a control arm. And to date only 6 of 61 PAPG students have not analyzed their own genome, an insufficient number to provide a meaningful comparison group for evaluating the differences in outcomes between those students who did and did not analyze their own genome.

Each year, however, there are many more interested students than we have funding to sequence. In response, starting in 2014 we offered waitlisted students the opportunity to enroll without the opportunity to obtain their genome or wait until next year; 5 such students enrolled and 3 completed the course. We plan to continue to expand enrollment in this way, both to satisfy student demand and to accumulate a meaningful comparison group.

Although PAPG students are “healthy”, PAPG was not a study of the impact on or clinical utility of WGS for healthy individuals. The outcomes of interest here were educational utility and psychological impact. The companion study did not collect other data, such as variants detected, that would have been relevant to studying clinical utility, nor do the authors attempt to extrapolate the experiences of PAPG students to other contexts. And conversely, WGS can have educational utility even if it is not cost-effective or widely used as a screening tool in healthy populations.

## Next steps

We believe it is critically important to offer hands-on training in NGS and genomic medicine, regardless of whether students analyze their own or reference genomes. As an indication of the need and utility, 17 of 21 students reported in the 2014 post-course survey that they had already applied the knowledge they gained to their course work, clinical practice or research.

Sequencing 20 whole-genomes a year, though, is expensive and not without the risk for test-related distress. Is it worth it? Unfortunately, as described above, we cannot yet quantitatively answer that question although the preliminary data is promising.

PAPG is one point on a broad spectrum of course offerings that include the PGT-based courses cited previously and many others. Another possible design could incorporate one of the many individuals who have obtained and publicly shared their own genome as a volunteer “case study”. This approach offers some of key features of PAPG; students are directly engaged with the complexity of genome sequencing and actively applying what they learn in real-world settings in which the results matter, but without the cost and complexity of incorporating student genomes. In the future, however, cost and complexity will be less of a barrier and the question will strictly be of the balance between the additional pedagogical value versus the potential adverse effects of analyzing your own genome.

The ultimate practice model around WGS is still to be determined. That we yet have a lot to learn is all the more reason to investigate different approaches for enhancing genomic education, including incorporating PGS [20]. We hope that this report will contribute to the discussion on how to most effectively provide much needed genomics education and motivate the development of new pedagogy to train the next generation of genomicists.

## Additional file

Additional file 1:
**Supplemental materials including the course approval process, course organization, additional lessons learned and course syllabi.** (PDF 200 kb)

## Abbreviations

WES: Whole exome sequencing
WGS: Whole genome sequencing
NGS: Next-generation sequencing
PGT: Personal genetic testing
