# Supplementary material for: Preparing the next generation of genomicists: a laboratory-style course in medical genomics
Source: BMC Med Genomics. 2015 Aug 12;8:47. doi: 10.1186/s12920-015-0124-y (PMC4534145; doi:10.1186/s12920-015-0124-y)
Supplement: Additional file 1: — Supplemental materials including the course approval process, course organization, additional lessons learned and course syllabi. (PDF 200 kb) [file 12920_2015_124_MOESM1_ESM.pdf]

Preparing the Next-Generation of Genomicists: A Laboratory-Style Course in Medical Genomics

Supplemental Materials

Michael D. Linderman[1,2], Ali Bashir[1,2], George A. Diaz[2], Andrew Kasarskis[1,2], Saskia C. Sanderson[1,2], Randi E. Zinberg[2], Sabrina Suckiel[1,2], Micol Zweig[1,2], Milind Mahajan[1,2], Hardik Shah[1,2], Eric E. Schadt[1,2]

[1]Icahn Institute for Genomics and Multiscale Biology, Icahn School of Medicine at Mount Sinai, One Gustave L. Levy Place, New York, NY 10029, USA.
[2]Department of Genetics and Genomic Sciences, Icahn School of Medicine at Mount Sinai, One Gustave L. Levy Place, New York, NY 10029, USA.

Table of Contents

1 Approval Process 2
2 Course Organization 2
  2.1 Informed Decision-Making Prior to Sequencing 4
  2.2 Sequencing Protocol 4
  2.3 Practical Analysis of Your Personal Genome Course 5
  2.4 Ongoing Monitoring and Post-course Actions 6
3 Lessons Learned 6
4 Introduction to Human Genome Sequencing Syllabus 9
5 Practical Analysis of Your Personal Genome Syllabus 11
6 References 13

1 Approval Process

The Practical Analysis of Your Personal Genome (PAPG) course and companion research study were initiated by the chair of the Genetics and Genomics Science (GGS) Department, and were reviewed by the following entities: the Dean of the Icahn School of Medicine, the CePORTED[1] Curriculum Committee, the Institutional Review Board (IRB), and the school's Research Ethics Committee during the spring and summer of 2012. We also consulted the medical center's general counsel and the New York State (NYS) Department of Health while designing the course.

The IRB determined that the purpose of the companion research study was a study of the student's decision-making process and feelings about analyzing about analyzing one's own genome in an educational setting, not a systematic investigation of the genome. Thus the sequencing component would not constitute research and would not be considered in their review of that study. The IRB then determined that the companion study was a survey-based minimal risk study and was thus exempt from informed consent requirements[1]. We point the interested reader to publications about the companion study[1, 2] for additional information about the study design.

The IRB director and assistant director referred the course directors to the institution's Research Ethics Committee for their review and approval of the sequencing component of the course, which was then obtained. The specific foci of committee's review were confidentially and coercion. The committee determined that those concerns were carefully and thoughtfully addressed by the mechanisms documented in the following section.

2 Course Organization and Student Population

The objectives of PAPG are summarized in **Error! Reference source not found.** and the structure and major content areas of PAPG are summarized in Figure 1. As described in the main text, the PAPG pedagogy was designed for current and future genetics professionals, including genetic counselors, medical and laboratory geneticists and research scientists. Table 2 lists the breakdown of student enrollment by year and background.

Table 1: Course objectives. At the conclusion of the course students should be able to:

1. Analyze a complete human genome sequence, starting with raw read data from 2nd-generation sequencing instruments through variant discovery and interpretation
2. Formulate hypotheses about the phenotypic significance of variants using public databases, literature and other resources
3. Communicate the results of a complete human genome analysis to others
4. Contribute to the public discussion about the ethical, legal and social implications of personal genomics

Our focus on the end-to-end analysis genome sequencing data reflects the increasingly blurry boundary between laboratory geneticists, who traditionally design and implement the genetic test, and the clinical providers who order the test and act on the results. Appropriately ordering and accurately interpreting the results of whole exome/genome

[1] Center for Patient Oriented Research, Training, Education and Development

sequencing (WES/WGS) and large next-generation sequencing-backed multi-gene tests requires a deep understanding of the capabilities and limitations of these technologies and the practical skills to analyze the more numerous, and potentially more ambiguous, findings.

Table 2: Percentage of students enrolled in PAPG by year and background/program

Percentage Enrolled	2012 (n=20)	2013 (n=19)	2014 (n=25)
**Genetic Counseling Students**	25%	42%	32%
**Graduate Students (incl. MD/PhD)**	25%	47%[1]	52%[2]
**Medical Genetics Residents**	20%	5%	12%
**Laboratory Geneticists**	10%	5%	4%
**Other**	20%	0%	0%

[1]Two of these students (10% of total) dropped the course during the semester
[2]Five (20% of total) enrolled without the opportunity to obtain their own genome, 3 of those 5 completed the course

The course and sequencing protocol are designed to maximize pedagogical value while mitigating the chance for coercion, maintaining privacy and mitigating the risk of test-related distress. These concerns and our mitigation strategies are summarized in Table 3. The details of the informed decision-making process, the sequencing protocol and the course organization are described in the following sections.

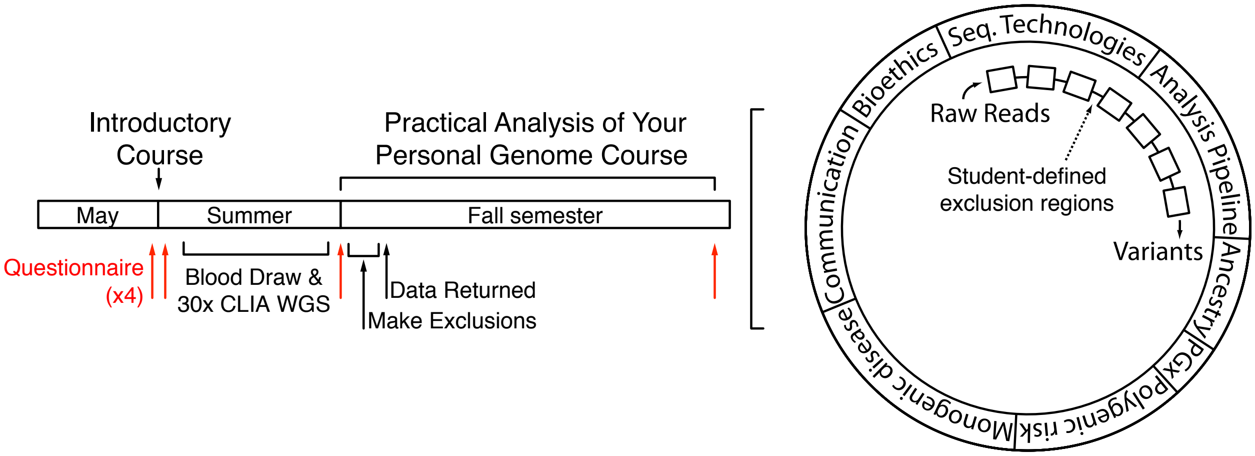

Figure 1: Organization and content of "Practical Analysis of Your Personal Genome" course

Table 3: Summary of concerns and mitigation strategies implemented

Concern	Mitigation Strategies
**Coercion**	1) Sequencing is optional<br>2) Instructors blinded to student choice
**Privacy**	1) Instructors blinded to student choice<br>2) Students exclusively control access to data
**Distress**	1) Introductory course to provide background information<br>2) One-on-one genetic counseling available free-of-charge<br>3) Implement exclusions during analysis

2.1 Informed Decision-Making Prior to Sequencing

Informed decision-making can be defined as the cognitive and emotional process that leads to a decision being made, when that decision is based on sufficient understanding and awareness of the risks, benefits, limitations, uncertainties and alternatives, and is consistent with the individual's attitudes or views[3].

A prerequisite for informed decision making in this context is sufficient understanding of whole genome sequencing (WGS) and the risk, benefits, uncertainties and limitations thereof. We developed a required prerequisite course, "Introduction to Human Genome Sequencing", which is effectively a condensed version of the longer PAPG course (the syllabus is included in Section 0). Most of the major content areas to be covered in PAPG are represented and students complete similar, albeit condensed, laboratory exercises with reference genomes. In 2012 this took the form of a 26-hour course spread over 2.5 weeks, in 2013 and onwards this course was condensed to a 17-hour workshop spread over two days (to facilitate scheduling). At the end of this course, the students had been exposed to many of the topics they would later encounter in PAPG, with a particular focus on the risks uncertainties and limitations of WGS. Surveys of the initial student cohort indicate that this course successfully reduced decisional uncertainty and decisional conflict[1].

We provide access to one-on-one genetic counseling, both within and outside the institution, free-of-charge, for students to discuss WGS in the context of their specific family medical history. One or more students utilized this service each year (self-reported), although in at least one case the motivation was simply to experience genetic counseling from a patient perspective[1]. We anticipated that students would discuss their decision with family members, friends, spouse/significant others, and or professional mentors and almost all students did[1]. We purposely schedule the blood draws several weeks after the completion of the introductory course to provide time for consultation and self-assessment.

Of particular concern in an educational context is that the student may feel pressured to analyze their personal genomes either directly or indirectly by their professors or peers, even if this goes against their wishes. Course instructors are blinded to the student's choice, and students are informed of this, to provide reassurance to students that there would be no negative educational repercussions to working with a reference genome. The students are instructed to say "my genome", regardless of their choice, so that their choice could not be inferred from how they discuss their data. And the students are asked not to inquire about their classmates' choices. All logistical issues related to the sequencing, such as indicating their choice to analyze their own data or not, are managed by a research coordinator separate from the course faculty. This coordinator serves as a broker to maintain student anonymity in all logistical questions that might intersect with their identity.

2.2 Sequencing Protocol

Students initiate the sequencing process by meeting with a research coordinator to review and sign the sequencing information sheet that was previously provided to them and complete the blood draw. Student genomes are sequenced in a CLIA-approved, CAP-accredited sequencing facility to approximately 30-fold mean coverage on an Illumina HiSeq using a 100 base-pair paired-end protocol. FASTQ files containing the raw

sequencing data, either the student's own genome or a gender-matched reference selected by an informatician, are made available to the students on the institutional shared computing cluster in a directory only the student can access.

Prior to receiving their sequencing data, students optionally prepare exclusion regions to prevent variant calling in portions of their genome that could contain information that they do not wish to learn. This process is modeled on the exclusions requested by James Watson, and the practical challenges in perfectly excluding unwanted information are discussed in detail during class[4].

The students are trained to run a fully automated genome analysis pipeline (GAP) that incorporates bwa, Picard, GATK, snpEff and other tools[5]. This pipeline has been validated for use in clinical genomic tests in NYS. The products of the pipeline are: 1) a VCF file containing all variant calls annotated with putative coding impact, frequency in 1000 Genomes, ESP and other public datasets, conservation, functional prediction and presence in OMIM, HGMD and other variant databases; 2) genetically-inferred ancestry; 3) comprehensive sequencing and variant quality information; and 4) an HTML-based report that summarizes the QC data, ancestry, PGx, polygenic disease risk for Type 2 Diabetes, Coronary Artery Disease, Age-related Macular Degeneration and Alzheimer's Disease (APOE ε4), reported disease mutations and rare coding variants in a set of approximately 600 known disease genes. None of these latter variants are interpreted for pathogenicity as part of this report. Students use Ingenuity Variant Analysis, PLINK/SEQ and custom-developed scripts to further analyze and interpret variants.

Students could change to working with a reference genome at any time during the course for any of the exercises.

2.3 Practical Analysis of Your Personal Genome Course

The PAPG course is organized into four modules: 1) exclusion generation, 2) variant discovery, 3) interpretation and communication, and 4) future technologies. The full syllabus can be found in Section 0. Variant discovery, interpretation and communication comprise the majority of the course and include: sequencing technology, short-read alignment and variant calling algorithms, ancestry, genome-wide association studies (GWAS) for physical traits and disease risk, genetically-informed risk prediction, variant interpretation in clinical contexts, and the communication of genetic information.

Each class session is structured as a lecture followed by hands on laboratory exercises using the student's genome or relevant reference data. Some example laboratory exercises include:

- Reviewing pileup to validate variant calls and identify structural variants
- Determine drug metabolizer phenotype using the star allele mapping provided by PharmGKB and relevant CPIC guidelines
- Interpret variants associated with physical traits, e.g. ability to taste PTC,
- Curate variants associated with common complex diseases from the GWAS literature, construct risk predictor using likelihood ratios[6] and compute post-test risk from genomic data and pre-test risk

- Group competition to build a Type 2 Diabetes genetic risk predictor. The different risk predictors are evaluated for discriminative accuracy in an ethnically diverse biobank.
- Classify pathogenicity of case-study variants using ACMG guidelines. Variants are sourced from the literature and ongoing sequencing projects, such as the Personal Genome Project
- Apply different filters to identify potential causal variants in a clinical case-study genome

2.4 Ongoing Monitoring and Post-course Actions

Those students who wish to clinically follow-up a particular finding, either during the course or afterwards, are encouraged to contact the genetic counselors who support the course during the decision-making phase or the clinical course instructors for referral to the relevant department or resource. The sequencing is performed in an educational, not clinical context, and so no findings are directly clinically actionable. Thus while the course may be the prompt for seeking clinical care, that care is ultimately distinct from the course, is obtained through existing clinical channels and would be the financial responsibility of the student.

The course instructors closely monitor the students for signs of distress. The school's mental health resources were notified about the course prior to its start so they could be prepared to assist students if needed.

The students have sole control over and access to their data and that remains unchanged after the course is completed. The institution supplied portable hard-drives at no charge to those students who were leaving the institution (and thus losing access to the institution's compute cluster) and wished to download their genomic data for future use. As reported in the companion study and directly observed by the instructors, students could and did continue to analyze their data after the course completed, either independently or through informal consultation with the course instructors.

3 Lessons Learned

For those groups considering offering a course like PAPG some of the lessons we have learned over the three iterations of PAPG are:

The uptake of personal genomes exceeded expectations and previous reports. Given their demonstrated interest in genomics, PAPG students are likely more interested in obtaining their own genome sequence than the general population. But there are also opportunity costs to declining the sequencing; WGS was 15-50 fold more expensive than DTC PGT used in similar courses and not readily available to PAPG students outside of this class. In the 2012 pre-course questionnaire the most endorsed perceived benefit (89%, n=19) for educational WGS was it would be "an opportunity that I would not ordinarily get if I had to pay full price"[2]. As WGS becomes more available the opportunity cost will shrink but in the meantime it is a potent motivator.

Few students engaged the formal one-on-one genetic counseling. As part of the informed decision-making process, we offered PAPG students optional one-on-one genetic

counseling at no cost to them. Only a few students scheduled a counseling appointment with the two affiliated counselors (one inside and one outside the institution). Many more students initiated discussions with the departmental genetic counselors who are not affiliated with the course, and in particular with the genetic counselor/research coordinator who was the student's logistical point-of-contact during the course. We observed this pattern with students who had pre-existing relationships with counselors and other students who did not.

As current and future genetics professionals, many of the PAPG students are well versed in the foundations of genetic counseling and thus may prefer informal conversations regarding their decision-making. To better support this mode of interaction, in future iterations of the course we plan to more widely disseminate information sheets about the course to better prepare departmental counselors for these conversations and create specific opportunities for more "informal" consultation with the genetic counselors who support the course.

We do not believe it is possible to eliminate the possibility for test-related distress when students analyze their own genome. To do so would require the impossible: perfect self-awareness and then perfect exclusion of unwanted information. To date the companion study[2] has identified one student who experienced a brief period of test-related distress when revisiting their data after the course. This distress was precipitated in part by the unanticipated discovery of a variant of unknown significance (VUS) in a gene associated with Brugada Syndrome, which the student subsequently determined to not be pathogenic without the involvement of the faculty. In a follow-up interview, this student reported being capable of interpreting that variant and aware that such a variant might be detected, but that actually doing so was distressing. This student said in that interview in the context of restricting the analysis to certain variants/genes (to attempt to prevent this kind of discovery) "You do have the choice, but I think sometime it's difficult to anticipate how you will react to a result that is not actionable"[2].

We are continually experimenting with new pedagogical tools to aid in self-assessment and an informed consideration of all possible results, not just those that are readily available with the current tools. This effort benefits from the ongoing discussion and scholarship around secondary findings[7] (all findings in healthy individuals are in effect "secondary") and informed decision-making more generally. Specifically, we: use the survey results collected during the development of the secondary findings guidelines[8] as a prompt for the students to consider different disease areas during decision-making and exclusion generation; are incorporating more "challenging" variants, e.g. VUS in genes with adult onset phenotypes, into course exercises; and are developing a set of "questions to consider", primarily elicited from the students themselves, for use during decision-making. We are in the process of evaluating the impact of these various efforts.

The scale of WGS can be overwhelming and the informatics tools a common stumbling block. Analyzing the 4-5 million variants identified in a whole genome requires numerous informatics tools and databases, many unfamiliar to the students, along with deep pathophysiological knowledge. We expected that the students would feel overwhelmed at times (as do the "experts"[9]), and the course evaluations bear that out. The purpose of this

class, though, is to directly confront and master the larger scale and complexity of these technologies and that is only possible through intensive and repeated hands-on experience.

PAPG's breadth ensured every student encountered new topics. A recurring theme, though, was difficulty with the informatics tools and concepts. The PAPG students typically did not have any prior informatics experience. Learning to use the various software tools was a necessary prerequisite for the laboratory exercises, but also sometimes a distraction from the core genomics concepts being taught; deciding which informatics details could be elided was a key pedagogical challenge. While NGS software tools have improved substantially since the initial iteration of PAPG, and will continue to do so, genomics will not stop being – in part – an informatics science; computational skills and algorithmic thinking will need to become a more integral part of genetics and genomics curricula at every level[10].

Many students self-disclose their sequencing choice, either purposely or implicitly through their questions. And when all of the students choose to sequence their own genome[1], their choice becomes known (although they could have later switched to a reference genome). One motivation for maintaining the privacy of the students' choice is to mitigate real or perceived pressure and students generally did not report feeling pressured by others when making their decision[1]. Additional study measures are needed, however, to assess the impact of the intimate class setting on the student's decisional satisfaction and response to any genetic results.

Maintaining the distinction between genome education and genome interpretation was challenging. The small class size and the overlapping roles of the faculty and students within the department can make it difficult to maintain the distinction between teaching and interpreting. Outside of the course, the students and faculty may be colleagues or even have a direct supervisory relationship. In those outside settings the instructors would routinely answer questions that she, the instructor, may avoid or answer indirectly in the context of the class when in respect to the student's genome. PAPG was an educational experience and not a substitute for a rigorous clinical evaluation. To maintain this distinction course instructors would answer questions about the process of interpreting a variant but deflect questions about interpreting that variant in the context of a student's health or family history. Differentiating between these two types of questions and then artfully deflecting the latter is, however, challenging to do in practice.

4 Introduction to Human Genome Sequencing Syllabus

Description:

Students in this hands-on laboratory-style workshop will analyze a publicly available whole human exome/genome, starting with raw sequencer output through clinical interpretation. Students will be introduced to the ethical, legal and social implications (ELSI) of personal genomics, the techniques and technologies for sequence analysis and the process of communicating those analyses to patients. This course is intended to provide an introduction to human genome sequencing for genetics professionals and trainees. At the conclusion of the class students will have the necessary background to make an informed decision about personal whole exome/genome sequencing.

Schedule:

Day 1: Morning – Introduction to Next-Generation Sequencing

Welcome and Introduction

Introduction to 2nd Generation Sequencing

Introduce the entire 2nd generation sequencing workflow, including the use of hybrid capture technology. Discuss the importance of CLIA and other certifications. Present limitations imposed by the sequencing technology itself.

Short-read Mapping and Calibration: FASTQs to BAMs

Introduce front-end of data pipeline for 2nd generation DNA sequencing technology including alignment and recalibration. Present commonly used read mapping algorithms and tools, and the strengths and limitations thereof.

Variant Calling: BAMs to VCF

Introduce back-end of data pipeline including variant calling for SNVs and indels and variant filtering. Call variants in genomic data. Focus on various sources of error in filtering, mapping and variant detection.

Introduction to Annotation

Review variant calling results with a focus on important quality metrics. Introduce tools and data resources used in annotating and interpreting a personal genome.

Day 1: Afternoon – Practical Session

Explore the results produced by the genome analysis pipeline in a hands-on session that includes:

1. Explore alignment results with particular attention to different error modes
2. Review QC metrics such a mean coverage, GC bias and quality-by-cycle
3. Review variants calls using the pileup and variant QC metrics.
4. Annotate variants of interest with data from 1000 Genomes Project and other sources with online tools such as Variant Effect Predictor.

Day 2: Morning – Genomics in the Clinic

Pharmacogenomics

Introduce pharmacogenomics using Warfarin and Clopidogrel as motivating examples.

Common Multi-factorial Disease Risk
Introduce techniques to estimating genetic risk for common multi-factorial disease using GWAS results from public databases, the literature and other resources.

Mendelian Disease
Build hypotheses of the nature of disease causing variant and translate those hypotheses into queries against the called variants. Introduce how variants could be prioritized for likely pathogenic effect.

Communicating and Responding to Genetic Results
Introduce how genetic testing results are communicated to patients, with particular focus on whole genome sequencing. Review current understanding of how patients make informed decisions about genetic testing and how they respond to genetic testing results emotionally and behaviorally.

Day 2: Afternoon – Practical Session

Practical Session
Analyze and interpret variants in different settings, including:

1. Determine recommended dosing for Warfarin based on relevant genotype data
2. Compute predicted risk for Type 2 Diabetes using GWAS data
3. Classify variant pathogenicity
4. Identify variants of interest in clinical case scenarios using example WES data

As a group discuss “questions to consider” during decision-making and issues related to the interpretation of the significance of genomic variants and how to communicate these findings.

5 Practical Analysis of Your Personal Genome Syllabus

Description:

Students in this course will analyze a human genome sequence starting with raw sequence reads through identifying a list of sequence variants. Using public databases, literature and other resources students will formulate hypotheses about the phenotypic significance of these variants. This is a hands-on, laboratory course in which students will choose to analyze either their own genome or a reference genome after lectures and counseling to make the consequences of personal genome analysis clear.

Prerequisites:

BSR2401, Introduction to Human Genome Sequencing

Expectations:

This is small, hands-on laboratory-style course and students are expected to be active participants and contributors to the class. Students will be evaluated according to:

20% Class Participation (including final group project)
20% Quizzes (4 randomly during the semester with the lowest dropped)
30% Homework
30% Capstone Project

Schedule:

Week	Topic
1	Questionnaire<br>Introduction to Minerva/HPC
1	Inclusion/exclusion criteria using ACMG guidelines, etc.; Reportability
2	Review human genetic variation and sources of disease/gene mappings<br>Assemble exclusion regions
2	Data return, initiate pipeline run
3	Bioethics applied to genomics
3	2nd Generation Sequencing; Tour Genome Core Facility
4	Sequence alignment
4	Variant calling and filtering (HW 1: Out)
5	Reviewing sequence QC; What is "good" sequencing?
5	Bogus results in medical genetics
6	Physical Traits
6	Common complex diseases; GWAS: How, what, why (HW 1: Due)
7	Applying GWAS results in personal genomics, part 1
7	Applying GWAS results in personal genomics, part 2
8	Pharmacogenomics
8	Mid-course Review
9	Monogenic disease
9	Strategies for variant classification (HW 2: Out)
10	Ancestry
10	DTC Genomics; Debate: What genetic information should be available?

11	Decision-making, communication, and behavioral change?
11	Participatory Genomics
12	Variant interpretation exercise, putting it all together (HW 2: Due)
12	Advanced Topics: Structural Variants
13	Advanced Topics: Cancer genomics, regulatory genomics
13	Group Work Period
14	Group Presentations
14	Capstone discussion<br>Questionnaire

6 References

1. Sanderson SC, Linderman MD, Kasarskis A, Bashir A, Diaz GA, Mahajan M, Shah H, Wasserstein M, Zinberg RE, Zweig M, Schadt EE: **Informed decision-making among students analyzing their personal genomes on a whole genome sequencing course: a longitudinal cohort study.** *Genome Med* 2013, **5**:113.

2. Sanderson SC, Linderman MD, Zinberg R, Bashir A, Kasarskis A, Zweig M, Suckiel S, Shah H, Mahajan M, Diaz GA, Schadt EE: **How do students react to analyzing their own genomes in a whole-genome sequencing course?: outcomes of a longitudinal cohort study**. *Genet Med* 2015.

3. Marteau TM, Dormandy E, Michie S: **A measure of informed choice**. *Heal Expect* 2001, **4**:99–108.

4. Nyholt DR, Yu C-E, Visscher PM: **On Jim Watson's APOE status: genetic information is hard to hide.** *Eur J Hum Genet* 2009, **17**:147–9.

5. Linderman MD, Brandt T, Edelmann L, Jabado O, Kasai Y, Kornreich R, Mahajan M, Shah H, Kasarskis A, Schadt EE: **Analytical validation of whole exome and whole genome sequencing for clinical applications.** *BMC Med Genomics* 2014, **7**:20.

6. Morgan AA, Chen R, Butte AJ: **Likelihood ratios for genome medicine.** *Genome Med* 2010, **2**:30.

7. Green RC, Berg JS, Grody WW, Kalia SS, Korf BR, Martin CL, McGuire AL, Nussbaum RL, O'Daniel JM, Ormond KE, Rehm HL, Watson MS, Williams MS, Biesecker LG: **ACMG recommendations for reporting of incidental findings in clinical exome and genome sequencing.** *Genet Med* 2013, **15**:565–74.

8. Green RC, Berg JS, Berry GT, Biesecker LG, Dimmock DP, Evans JP, Grody WW, Hegde MR, Kalia S, Korf BR, Krantz I, McGuire AL, Miller DT, Murray MF, Nussbaum RL, Plon SE, Rehm HL, Jacob HJ: **Exploring concordance and discordance for return of incidental findings from clinical sequencing.** *Genet Med* 2012, **14**:405–10.

9. Chrystoja CC, Diamandis EP: **Whole genome sequencing as a diagnostic test: challenges and opportunities.** *Clin Chem* 2014, **60**:724–33.

10. Association of American Medical College, Howard Hughes Medical Institute: *Scientific Foundations for Future Physicians*. 2009.
